# Enhancing PrEP adherence through person‐centred mobile app interventions: a real‐world data and machine learning approach using UPrEPU among gay, bisexual and other men who have sex with men in Taiwan

**DOI:** 10.1002/jia2.70033

**Published:** 2025-10-08

**Authors:** Jay Chiehen Liao, Huei‐Jiuan Wu, Tsan‐Tse Chuang, Tsai‐Wei Chen, Carol Strong

**Affiliations:** ^1^ Department of Public Health College of Medicine National Cheng Kung University Tainan Taiwan; ^2^ Department of Computer Science and Information Engineering College of Electrical Engineering and Computer Science National Taiwan University Taipei Taiwan; ^3^ Kirby Institute UNSW Sydney Sydney New South Wales Australia

**Keywords:** pre‐exposure prophylaxis, gay bisexual and other men who have sex with men, adherence, machine learning, Taiwan

## Abstract

**Introduction:**

Pre‐exposure prophylaxis (PrEP) is an effective HIV prevention tool that relies on good adherence in high‐risk scenarios. To understand the factors that predict adherence, technology such as mobile applications like UPrEPU—allowing for logging users’ daily behaviours at close to the time they have sex or PrEP intake—can be used as a person‐centred, self‐care intervention. This study aims to develop a machine learning model using logs of sexual activities and user attributes recorded in the UPrEPU mobile application in Taiwan to predict whether a sexual event was protected by oral PrEP among gay, bisexual and other men who have sex with men (GBMSM).

**Methods:**

We used data from the UPrEPU app collected between January 2022 and May 2023 in Taiwan. The dataset included information on users’ sex events, such as the timing and users’ sex roles (e.g. versatile, receptive or insertive partner), and the dynamic user‐based attributes related to sexual behaviours and PrEP use. Various subsets of these features were employed in CatBoost models to predict whether the sex events were associated with correct PrEP use. We evaluated the models’ performance using five‐fold cross‐validation. The influential features were identified through feature importance analysis and Shapley Additive Explanations (SHAP) values to explain the models.

**Results:**

A total of 198 users recorded 2356 anal sex events on UPrEPU. The model with dynamic user‐based attributes outperformed those without them. The most parsimonious model had a good prediction performance (accuracy = 75%, precision = 78%, recall = 90%, F1‐score = 83%) and identified the key features of PrEP protection. The model with five dynamic user‐based attributes—age, cumulative PrEP use, condom use and the proportion of anal sex events with HIV‐negative partners not on PrEP—significantly outperformed the model based on event‐level attributes alone.

**Conclusions:**

Behavioural patterns significantly influence PrEP adherence among GBMSM. Person‐centred mobile applications such as UPrEPU provide valuable data for tailored, just‐in‐time interventions, enhancing adherence. Recognizing these patterns can guide person‐centred interventions. Incorporating these insights into clinical care or digital tools may improve consultations and support timely, informed HIV prevention decisions.

## 1 INTRODUCTION

Oral HIV pre‐exposure prophylaxis (PrEP) is highly effective but requires adequate adherence. Alongside daily dosing, event‐driven PrEP—added to Taiwan's guidelines in 2018 [1] – has gained popularity among gay, bisexual and other men who have sex with men (GBMSM) in western Europe, Australia and Taiwan, the focus of this study [2, 3, 4, 5]. Event‐driven PrEP involves two pills between 2 and 24 hours before sex, then one daily until 48 hours after the last sexual encounter. However, a prospective cohort study of GBMSM in Taiwan found that adherence to event‐driven PrEP may be challenging due to unexpected sexual situations, lack of opportunity to plan in advance and the regimen's complexity [4]. While overall adherence was high, switching from daily to event‐driven PrEP was associated with poorer adherence [4]. Conventional self‐report tools often miss episodic behaviours, while daily diaries using ecological momentary assessment (EMA) offer context into sexual behaviours [6].

We, therefore, leveraged EMA data from UPrEPU, a self‐monitoring system designed to monitor and improve adherence to event‐driven and daily HIV PrEP among GBMSM in Taiwan [7]. Developed with user‐centred design incorporating user input, the app accommodates complex dosing behaviours linked to sexual practices and dynamic dosing choices, and allows users to log behaviour close to the time of sex or PrEP intake. To enhance adherence, the app used the calendar feature to track the user's dosing regimen and scheduled sexual activity, sending push notifications for upcoming pill doses accordingly. UPrEPU app supported 68.6% correct PrEP use during anal sex events, with users reporting it was helpful for self‐management [7].

Our objective was to develop machine learning models to predict PrEP protection using logs of sexual activities and user attributes data. We examined whether adding user‐level attributes improves PrEP protection prediction and identified key behavioural and contextual predictors. The use of predictive models informed by individual data fosters a more person‐centred approach to PrEP adherence by providing targeted support and enhanced monitoring.

## 2 METHODS

### 2.1 Study design and setting

We conducted a retrospective analysis of data from individuals enrolled in the UPrEPU application, publicly available in Taiwan since 1 January 2022 (732 downloads up to May 2023).

### 2.2 Study participants

Eligible participants were assigned male at birth, aged ≥20, identified as GBMSM and provided informed consent to use the app for logging PrEP intake or sexual activity. Those self‐reporting HIV‐positive status at enrolment were excluded. For users who later reported seroconversion, only pre‐seroconversion data were analysed. Inclusion also required at least one logged PrEP or sexual activity event.

### 2.3 Variables

Data in the UPrEPU app from January 2022 to May 2023 were included in the present study, including users’ demographic attributes and logs of their PrEP use and anal sex events.

### 2.4 Outcome

Our primary outcome is a binary variable indicating whether PrEP was taken correctly for an anal sex event. A correct intake was determined if one of the following two criteria was satisfied:

1. For at least 4 out of 7 days before a sexual event, at least one pill was taken each day.
2. Two pills of PrEP were taken between 2 and 24 hours before, followed by one pill taken between 24 and 48 hours, respectively, after the first pill intake. However, if at least one pill was taken within 7 days before each sexual event, taking only one pill 2–24 hours before each sexual event was allowed.

### 2.5 Predictors and machine learning model choice

We categorized all predictors involved in this study into two groups:

- Event‐based attributes: characteristics of each sexual event, such as the user's sex role, timing of the event and condom use.
- User‐based attributes: personal characteristics, primarily derived from cumulative or average data across events, such as the cumulative number of anal sex events, the cumulative number of times PrEP was taken and the proportion of condom use up to the current event.

### 2.6 Training, evaluation and explanation of machine learning models

We used CatBoost to construct our machine learning models. Among the models tested (see code and results on GitHub: https://github.com/jayenliao/uprepu‐prep‐adherence‐ml), CatBoost was selected for its significantly better performance compared to Random Forest and XGBoost in our empirical evaluation [8, 9, 10]. It is particularly well‐suited to our data, as it natively handles categorical features using permutation‐based target statistics and accommodates missing values through sentinel imputation, eliminating the need for normalization or scaling [8].

Two CatBoost classifiers were constructed:

1. Model 1 (M1): All 11 event‐based attributes.
2. Model 2 (M2): Besides 11 event‐based attributes, 19 dynamic user‐based attributes with contextual information were also included, resulting in 30 features. Five nested variants of M2 (M2‐a through M2‐e), containing successively fewer features (i.e. 25, 20, 15, 10 and 5), were also evaluated.

To prevent overfitting and respect the temporal ordering of events, we employed a temporal 10‐fold cross‐validation scheme, thereby avoiding peeking into the future. The full dataset was sorted by sex and event timestamp and split into 11 consecutive time‐based blocks. For each fold (i = 1, …, 11), training was performed on blocks 1 through i, and testing was performed on block i+1. This procedure yields 10 train–test splits that preserve temporal integrity and prevent information leakage. For each split, we computed accuracy, precision (i.e. positive predictive value, PPV), recall (i.e. sensitivity) and F1‐score, and reported their means and standard deviations across folds [11]. All hyperparameters were set to their default values. To test whether M1 and M2 perform significantly, we applied Wilcoxon signed‐rank tests to the F1‐scores observed in folds [12]. The F1‐score is the harmonic mean of the precision and recall scores and thus contains information about both the positive predictive value and the sensitivity [13].

Feature importance values extracted from the best‐performing model were used to identify and eliminate the five least influential attributes, resulting in more parsimonious variants with similar predictive power [14]. Wilcoxon signed‐rank tests comparing M2 with its reduced‐feature variants (M2‐a through M2‐e) confirmed no significant performance differences, and the code (including model comparison table) is available at: https://github.com/jayenliao/uprepu‐prep‐adherence‐ml. Finally, we employed Shapley Additive Explanations (SHAP), a game‐theory–based framework, to quantify the direction and magnitude of each feature's contribution to the predicted probability [15]. All analyses were conducted using Python software version 3.9.10.

### 2.7 Ethical approval

Ethical approval was granted by the Institutional Review Board of National Cheng Kung University Hospital [A‐ER‐110‐201]. Participants were not required to provide names when using the app. The app included explicit consent prompts. Data was securely stored, encrypted during transmission and protected against unauthorized access.

## 3 RESULTS

### 3.1 Cohort characteristics

From January 2022 to May 2023, 198 users in Taiwan recorded 2356 anal sexual events on UPrEPU. On average, the users spent 155 days and 19.5 hours on the application. The average number of sexual events for each person was 11.9 (standard deviation 19.8), and the median was 4.5. Among all anal sex events, 1662 (70.5%) were protected with the correct PrEP use based on our definition. Descriptive statistics of the demographic attributes and other variables included in the models are presented in Table 1.

**Table 1:** Descriptive statistics of event‐based and user‐based variables used in predictive models of PrEP adherence (*N* = 2356 anal sex events recorded by 198 participants)

Name and description	Mean (SD)/*N* (%)^a^
**(a) Event‐based (included in M1 and M2)**	
**Time of the sex event**: hour of day, 24‐hour format, that is 0, 1, . . ., 23.	15.0 (7.3)
**Pre‐date**: Whether the user had created the dating record before sex time for the current anal sex event.	215 (9.1%)
**Sex role**: User's sex role in the event	
*Receptive partner*	1203 (51.1%)
*Insertive partner*	908 (38.5%)
*Versatile*	245 (10.4%)
**Mood**: User's reported mood in the event	
*Terrible/bad/so‐so*	579 (24.6%)
*Good*	987 (41.9%)
*Fabulous*	790 (33.5%)
**Condom**: Whether a condom was used during the event	491 (20.8%)
**Icon default**: Whether the user set a default icon for the partner in the current event	1738 (73.8%)
**Partner age**: Age group of the partner in the event	
*Younger than 21*	252 (10.7%)
*21–30*	980 (41.6%)
*31–40*	876 (37.2%)
*41–50*	220 (9.3%)
*51–60*	21 (0.9%)
*Older than 60*	7 (0.3%)
**Older partner**: Whether the partner in the event was older than the user	568 (24.1%)
**Younger partner**: Whether the partner in the event was younger than the user	719 (30.5%)
**Same‐age partner**: Whether the user and the partner in the event belonged to the same age group	1069 (45.4%)
**Partner HIV status**: The HIV status of the partner in the event	
*Unknown*	1282 (54.4%)
*Negative and on PrEP*	449 (19.1%)
*Negative but not on PrEP*	458 (19.4%)
*Positive and undetectable*	124 (5.3%)
*Positive and viral load unknown*	9 (0.4%)
Missing	34 (1.4%)
**(b) User‐based (included in M2)**	
**User duration**: How long has the participant joined in the study up to the current event (unit: hour)	137.7 (110.7)
**Age**: How old the participant was at the time of the current anal sex event (unit: year)	31.2 (5.8)
**Sex cumulative sum**: The cumulative number of anal sex events the participant has recorded up to the current event.	22.9 (30.4)
**Date cumulative sum**: The cumulative number of dating records the participant has recorded up to the current event.	23.0 (30.5)
**Dose cumulative sum**: The cumulative times the participant has taken PrEP up to the current event.	62.7 (68.5)
**Switch cumulative sum**: The cumulative times of switching the regimen of taking PrEP the participant has conducted up to the current event.	0.6 (1.4)
**Condom use proportion**: Proportion of condom use up to the current event.	0.2 (0.3)
**Pre‐date proportion**: Proportion of dating records created on the app before sex up to the current event.	0.1 (0.2)
**Receptive proportion**: Proportion of being the receptive partner during anal sex up to the current event.	0.4 (0.4)
**Insertive proportion**: Proportion of being the insertive partner during anal sex up to the current event.	0.5 (0.4)
**Versatile proportion**: Proportion of being versatile during anal sex up to the current event.	0.1 (0.2)
**Older partner proportion**: Proportion of having older partner(s) up to the current event.	0.2 (0.3)
**Younger partner proportion**: Proportion of having younger than partner(s) up to the current event.	0.3 (0.3)
**Same‐age partner proportion**: Proportion of belonging to the same age group of partner(s) up to the current event.	0.5 (0.3)
**HIV‐unknown partner proportion**: Proportion of having anal sex with a partner whose HIV status was unknown.	0.5 (0.4)
**HIV– & PrEP+ proportion**: Proportion of having anal sex with a partner who was HIV negative and on PrEP.	0.2 (0.3)
**HIV– & PrEP– proportion**: Proportion of having anal sex with a partner who was HIV negative and not on PrEP.	0.2 (0.3)
**Undetectable HIV+ partner proportion**: Proportion of having anal sex with a partner living with HIV with undetectable viral load.	0.0 (0.1)
**HIV+ & viral load unknown partner proportion**: Proportion of having anal sex with a partner living with HIV with unknown viral load.	0.00 (0.02)

Abbreviations: HIV–, HIV‐negative status; HIV+, HIV‐positive status; M1, model 1, the CatBoost classifier with all 11 event‐based attributes; M2, model 2, the CatBoost classifier with 11 event‐based attributes and 19 dynamic user‐based attributes; PrEP, pre‐exposure prophylaxis; PrEP+, on PrEP; PrEP–, not on PrEP.

^a^ Values are generally reported to one decimal place. For very small proportions (< 0.05), two decimals are shown to avoid rounding to zero.

### 3.2 Machine learning models

Table 2 presents the testing performance of models with different feature sets and two prediction targets, evaluated using four indices. The positive label (i.e. the protected event) is our primary target for prediction. We also presented the negative label (i.e. the unprotected event) for reference due to label imbalance, as the proportion of protected events was much higher than its counterpart (i.e. 70.5% vs. 29.5%). This imbalance naturally drives lower scores for the minority class (i.e. the unprotected event).

**Table 2:** The performance of models with different feature sets and two prediction targets regarding four evaluation indices: accuracy, precision, recall and F1‐score (averaged scores of 10 folds ± standard deviations; *N* = 2356 anal sex events recorded by 198 participants)

Model^a^	Prediction target^b^	Accuracy^c^	Precision^d^	Recall^e^	F1‐score^f^
M1: CatBoost with 11 features	Protected	0.70 ± 0.05	0.73 ± 0.03	0.92 ± 0.08	0.81 ± 0.04
	Unprotected		0.43 ± 0.19	0.15 ± 0.09	0.21 ± 0.11
M2: CatBoost with 30 features	Protected	0.76 ± 0.05	0.79 ± 0.03	0.91 ± 0.07	0.84 ± 0.04
	Unprotected		0.64 ± 0.13	0.40 ± 0.10	0.48 ± 0.10
M2‐a: CatBoost with 25 features	Protected	0.76 ± 0.05	0.79 ± 0.02	0.90 ± 0.07	0.84 ± 0.04
	Unprotected		0.62 ± 0.14	0.39 ± 0.10	0.48 ± 0.11
M2‐b: CatBoost with 20 features	Protected	0.76 ± 0.05	0.79 ± 0.02	0.91 ± 0.06	0.84 ± 0.04
	Unprotected		0.65 ± 0.12	0.40 ± 0.09	0.49 ± 0.09
M2‐c: CatBoost with 15 features	Protected	0.76 ± 0.05	0.79 ± 0.02	0.90 ± 0.08	0.84 ± 0.04
	Unprotected		0.63 ± 0.14	0.39 ± 0.11	0.47 ± 0.10
M2‐d: CatBoost with 10 features	Protected	0.75 ± 0.05	0.78 ± 0.02	0.89 ± 0.09	0.83 ± 0.05
	Unprotected		0.63 ± 0.13	0.37 ± 0.10	0.45 ± 0.09
M2‐e: Catboost with 5 features	Protected	0.75 ± 0.05	0.78 ± 0.02	0.90 ± 0.07	0.83 ± 0.04
	Unprotected		0.61 ± 0.14	0.35 ± 0.08	0.44 ± 0.09

Abbreviations: M1, model 1; M2, model 2.

^a^ Model 1 (M1) includes all 11 event‐based attributes. For Model 2 (M2), besides 11 event‐based attributes, 19 dynamic user‐based attributes with contextual information were also included, resulting in 30 features. M2‐a, M2‐b, M2‐c, M2‐d and M2‐e are M2's variants with features reduction.

^b^ The prediction target can be either a protected or an unprotected event. Evaluation metrics are calculated accordingly based on the chosen prediction target.

^c^ Accuracy is the proportion of correctly predicted events (whether protected or unprotected) out of all events.

^d^ Precision (i.e. Positive Predictive Value, PPV) is the proportion of correctly predicted events of the target type (e.g. unprotected) among all events predicted as that type.

^e^ Recall (i.e. Sensitivity) is the proportion of actual events of the target type (e.g. protected) that were correctly identified by the model.

^f^ F1‐score is the harmonic mean of precision and recall, representing a balance between the two.

The Wilcoxon signed‐rank test showed that M2 significantly outperformed M1 with a higher F1‐score (*p* < 0.005), underscoring the value of incorporating user‐based attributes with contextual information into the prediction model.

No significant difference was observed between M2 and its variants. Exclusion of the five least important features (yielding M2‐b through M2‐e) did not materially affect performance (*p* > 0.05), indicating that the top predictors alone capture the majority of the signal for protected‐event prediction. Among M2's variants, M2‐e, its most parsimonious form, comprising only five features and proves sufficiently powerful to predict correct PrEP use, with all evaluation metrics remaining on par with those of more complex variants.

**Figure 1:**
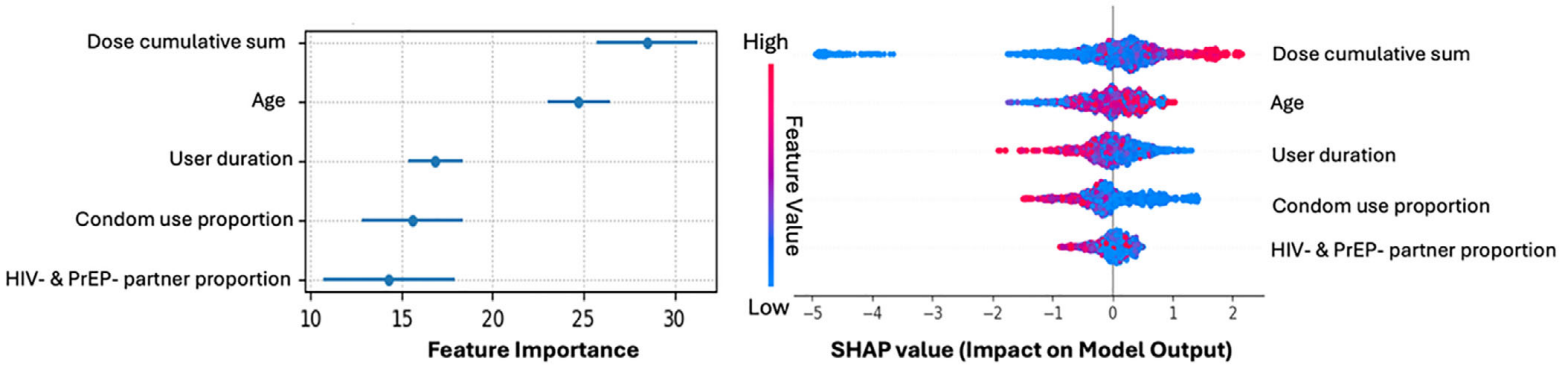
(a) Plot of feature importance of M2‐e. (b) Summary plot of SHAP value of M2‐e. M2‐e is the CatBoost model with five features (*N*=2356 anal sex events recorded by 198 participants). In Figure (a), dots indicate the averaged importance values, and error bars are the standard deviation among 10 folds of cross‐validation. In Figure (b), each sexual event is represented by a dot in each feature row. Dots in deeper red represent higher feature values, while those in deeper blue indicate lower values. Negative SHAP values have a negative impact on the final prediction probabilities, and vice versa. Abbreviations: Age, sex event time minus user's birthday; Condom use proportion, proportion of using condom of the user up to the current event; Dose cumulative sum, cumulative total count of times of taking PrEP; HIV– and PrEP– proportion, proportion of having anal sex events with a partner who was HIV negative and not on PrEP; M2, model 2; PrEP, pre‐exposure prophylaxis; SHAP, Shapley Additive Explanations; User duration, sexual event time minus user's time of APP registration.

Figure 1 presents five variables included in the M2‐e, namely dose cumulative sum, age, user duration, condom use proportion, and proportion of having anal sex events with a partner who was HIV negative and not on PrEP, and their feature importance and SHAP values. Among the five variables, the dose cumulative sum has the highest feature importance. The SHAP value indicated that the duration of using UPrEPU and the proportion of condom use were negatively associated with correct PrEP use.

## 4 DISCUSSION

Correct PrEP use was closely linked to each individual's behavioural patterns, which were influenced by their habitual tendencies and personal choices in the Taiwanese context. The model with five dynamic user‐based attributes—age, cumulative PrEP use, condom use and the proportion of anal sex events with HIV‐negative partners not on PrEP—significantly outperformed the model based on event‐level attributes alone. Understanding these patterns can enhance predictive models and support person‐centred interventions. Integrating this insight into clinical care or digital tools may improve consultations and support timely, informed HIV prevention decisions.

The cumulative number of taking PrEP was the most influential factor, suggesting that adherence may involve a learning curve in recognizing behaviours and risks to adopt more effective prevention strategies. This finding aligns with prior research showing that frequent self‐monitoring and visual feedback through mobile apps can reinforce PrEP adherence by enhancing users’ ability to recognize behavioural patterns and implement timely prevention strategies [16, 17].

Our study indicated that certain longitudinal behavioural patterns within a person are indicative of correct PrEP use, such as cumulative condom use and proportion of sex encounters involving HIV‐negative partners not on PrEP. Such longitudinal data are important because prior research on HIV risk factors and PrEP adherence has examined both individual characteristics and contextual factors. Situational variables, such as weekends or unusual days, were strongly associated with non‐adherence in several studies [6, 18, 19], while fluctuating emotional or motivational states have been tied to higher‐risk sexual activity, including condomless anal intercourse [6]. A valuable next step could be the collection of psychosocial attributes and clinical characteristics through the app and utilizing those in the machine learning models.

Interestingly, we observed that lower rates of condom use indicated a higher probability of correct PrEP use during sexual encounters among users. This suggests the importance of considering both prevention and adherence strategies simultaneously when evaluating HIV prevention approaches in real‐world settings, which echo the importance of preventive‐effectiveness adherence [20].

Our study indicated that the longer duration of using UPrEPU is negatively associated with reported correct PrEP use, possibly due to declining engagement with the app's logging features over time. This highlights the need to distinguish between the app as an adherence support tool and as a self‐report platform. Given the limitations of self‐reported data, future studies using objective biomarkers like drug concentrations could provide more accurate adherence measures. Sustaining engagement remains a challenge, as seen with other self‐logging health apps [21, 22, 23], and automated solutions such as Internet of Things integration may help reduce user burden.

The present study has the following limitations. The major limitation of the study is non‐response bias, such as not reporting all sexual activities or PrEP use on the app. Individuals who are keener on actively monitoring and logging their behaviour in the app might be those with better adherence. Further, an assumption is made that most individuals engage in planned sexual activity, allowing our app to remind them to take medication in advance. However, for those primarily involved in unplanned sexual encounters, this assumption may not hold, potentially leading to decreased adherence and incomplete or inaccurate adherence patterns. Lastly, our analysis may have potential over‐representation bias since we did not differentiate inter‐ and intra‐participant variability. The model results may be dominated by a few users who have much frequent logins than others.

## 5 CONCLUSIONS

Our study explores the predictive value of sexual behavioural patterns in PrEP adherence in Taiwan, highlighting the need for person‐centred approaches to strengthen prevention strategies and support timely, informed decisions.

## COMPETING INTERESTS

The authors declare no competing interests.

## AUTHOR CONTRIBUTIONS

JCL conceived the presented research idea and verified the underlying data. JCL, T‐TC and T‐WC conducted the data collection, laboratory activities, and reviewed the collected data for quality and reliability. JCL analysed the data and constructed machine learning models. JCL, H‐JW and CS contributed to interpreting the results and took the lead in writing the manuscript. CS was in charge of the overall direction and planning. All authors provided critical feedback, shaped the research, analysed the manuscript and approved the final submitted manuscript.

## FUNDING

The study was funded by grants from the Taiwan Ministry of Science and Technology (MOST 111‐2636‐B‐006‐011, NSTC 112‐2636‐B‐006‐007, NSTC 112‐2314‐B‐006‐081‐MY3).

## Data Availability

The data that support the findings of this study are available from the corresponding author upon reasonable request.
